# Promoting water consumption among children: a three-arm cluster randomised controlled trial testing a social network intervention

**DOI:** 10.1017/S1368980020004802

**Published:** 2021-06

**Authors:** Crystal R Smit, Rebecca NH de Leeuw, Kirsten E Bevelander, William J Burk, Laura Buijs, Thabo J van Woudenberg, Moniek Buijzen

**Affiliations:** 1 Behavioural Science Institute, Radboud University, Nijmegen, The Netherlands; 2 Erasmus School of Social and Behavioural Sciences, Erasmus University Rotterdam, Rotterdam, The Netherlands; 3 Radboud Institute for Health Sciences, Radboud University and Medical Centre, Nijmegen, The Netherlands

**Keywords:** Social network intervention, Peer influence, Social norms, Water, Sugar-sweetened beverages

## Abstract

**Objective:**

To test the effectiveness of a social network intervention (SNI) to improve children’s healthy drinking behaviours.

**Design:**

A three-arm cluster randomised control trial design was used. In the SNI, a subset of children were selected and trained as ‘influence agents’ to promote water consumption–as an alternative to sugar-sweetened beverages (SSB)–among their peers. In the active control condition, all children were simultaneously exposed to the benefits of water consumption. The control condition received no intervention.

**Setting:**

Eleven schools in the Netherlands.

**Participants:**

Four hundred and fifty-one children (*M*_age_ = 10·74, SD_age_ = 0·97; 50·8 % girls).

**Results:**

Structural path models showed that children exposed to the SNI consumed 0·20 less SSB per day compared to those in the control condition (*β =* 0·25, *P* = 0·035). There was a trend showing that children exposed to the SNI consumed 0·17 less SSB per day than those in the active control condition (*β =* 0·20, *P* = 0·061). No differences were found between conditions for water consumption. However, the moderation effects of descriptive norms (*β* = –0·12, *P* = 0·028) and injunctive norms (*β* = 0·11–0·14, both *P* = 0·050) indicated that norms are more strongly linked to water consumption in the SNI condition compared to the active control and control conditions.

**Conclusions:**

These findings suggest that a SNI promoting healthy drinking behaviours may prevent children from consuming more SSB. Moreover, for water consumption, the prevailing social norms in the context play an important role in mitigating the effectiveness of the SNI.

The prevalence of overweight and obesity in children remains a major global health concern^(1)^. The consumption of sugar-sweetened beverages (SSB) has been identified as a significant contributor to weight gain in children^(2)^. Reducing the consumption of SSB can be an effective strategy for the prevention of childhood overweight and obesity^(3)^. In particular, promoting water consumption as an alternative to SSB seems to be a promising approach^(4)^. Mass media campaigns are widely used in the public health sector to address excessive SSB consumption^(5–7)^. In these campaigns, large populations are simultaneously exposed to health messages in a rapid manner through various media channels^(8)^. Unfortunately, with such campaigns, the overall average behavioural change occurs in only 8% of the population^(9)^. A possible reason for their limited effectiveness could be that these mass campaigns, among others, do not incorporate the strong influence of peers^(10,11)^. Therefore, the current study investigated whether an approach that utilises peer influence can be more effective in promoting healthy drinking behaviours among children.

State-of-the-art intervention studies promoting other health-related behaviours, such as fruit and vegetable consumption^(12)^, physical activity^(13)^, condom use^(14)^ and smoking cessation^(15,16)^, revealed that utilising peer influence can be beneficial in promoting healthy behaviours. In these so-called ‘social network interventions (SNI)’, the influence of peers is utilised by selecting a subset of children as influence agents to diffuse the target health message or behaviour into the children’s network^(17,18)^. At the heart of this approach lies the diffusion of innovation theory^(19)^, which describes how new ideas and behaviours are spread among members of a social network. During the diffusion process, some individuals (i.e. influence agents) have more influence on the behaviour of others due to their unique position in the network^(20)^. Deploying these influence agents as advocates of the target behaviour (e.g. as role models) can accelerate the diffusion process and behaviour change in social networks^(21)^.

There is promising evidence from recent pilot studies that children’s drinking behaviour can be improved with such a social network-based approach^(22,23)^. In these studies, the influence agents were trained to encourage water consumption–as an alternative to SSB–among their peers. In both studies, an increase in children’s water consumption, as well as a decrease in their SSB consumption, was found^(22,23)^. However, these studies only investigated the effectiveness of the SNI by comparing it to a control condition. Thus, the question remains whether this promising social network-based approach is actually more effective than an active control condition based on the principles of mass media campaigns.

Moreover, SNI utilising peer influence are assumed to tap into normative behaviours. Research has shown that children do not like to deviate from the group norms and experience a strong need for acceptance, which prompts them to conform to the normative behaviour of their peers^(24–26)^. The literature distinguishes between two types of social norms, namely descriptive and injunctive norms^(27,28)^. Descriptive norms refer to the perception of how most people behave^(28)^. For healthy drinking, for example, this would imply that children perceive that their peers drink a certain amount of water. Injunctive norms refer to the perception of what others consider appropriate^(28)^. For example, an injunctive norm for healthy drinking would be that children perceive approval of their peers when they drink a certain amount of water. Several studies have shown that both type of norms affect children’s dietary behaviours with regard to the type and amount of food they perceive their peers to consume or approve of^(26,29–31)^. Therefore, it is conceivable that the success of peer-led interventions may depend on the prevailing social norms in the target network.

As yet, only one SNI included the moderating role of social norms, finding that the children’s injunctive norms interacted with the success of the SNI promoting water consumption^(22)^. That is, children who initially perceived high injunctive peer norms to consume water reported an increase in their water consumption^(22)^. In this case, the promoted behaviour in this intervention was in accordance with the norm children perceived beforehand. It is, therefore, plausible that SNI are more successful for children who perceive that the prevailing norm is in accordance with the promoted behaviour. Nevertheless, it is also plausible that SNI are more effective for children who initially perceived a discrepancy between the prevailing norm and the promoted behaviour. When the desired behaviour is promoted in the intervention, it could be that they want to live up to the promoted norm and adjust their behaviour accordingly. Thus far, there has been only one study that showed that the success of SNI depends on the prevailing injunctive norms and none on descriptive norms. Given the sparse research attention so far, this study explored the moderating role of both descriptive and injunctive norms.

Thus, the current study tested whether an intervention utilising peer influence was more effective than an active control condition – based on the principles of mass media campaigns – and a control without any intervention. We also investigated the moderating role of the prevailing social norms in the context. We hypothesised that children who were exposed to the SNI promoting water consumption as an alternative to SSB would report consuming more water post-intervention than those in the active control condition (H1a) and control condition (H1b). We also expected that children who were exposed to the SNI would report consuming less SSB post-intervention than those in the active control condition (H2a) and control condition (H2b). Finally, we explored the moderating role of descriptive and injunctive norms on the effectiveness of the SNI.

## Methods

### Design

The study involved a three-arm cluster randomised controlled trial with schools as the unit of randomisation. The schools were randomly assigned to either the (1) existing SNI (called *Share H*_*2*_ *O*), (2) active control condition or (3) control condition by a random allocation algorithm. In the SNI, children were exposed to peers from their own classroom who were identified and trained as influence agents to promote water consumption as an alternative to SSB consumption. The active control condition was based on the principles of mass media campaigns and, therefore, consisted of exposing all children simultaneously to a presentation on the benefits of water consumption. Children in the control condition did not receive any intervention. The required sample size was based on the previous pilot study^(23)^ where a small effect of the SNI was found with 210 children with a SNI condition and control condition. This number was multiplied with 1·5 to add the third condition (i.e. active control) in the current study, resulting in a minimum number of 315 children across the 3 groups. In order to take non-response in the active consent procedure into account, a larger number of children were recruited.

### Procedure

The study took place from February to June 2018 and consisted of three assessments: baseline (February–March 2018; T1), immediately after the start of the intervention (April–May 2018; T2) and during a follow-up 4 weeks later (June–July 2018; T3). At each assessment, children received a smartphone with a pre-installed research application and an activity-tracking bracelet for 7 days^(32,33)^. Via the research application, children received questionnaires and were also able to chat and to share pictures and short videos with peers. At T1, children completed drinking-related measures and sociometric nominations. Identical measures were assessed at T2 and T3. To assess whether children were aware of the actual purpose of the study, they were asked at T3 to describe what they believed to be the purpose of the study. None of the children indicated that the goal of the study was for influence agents to promote water consumption.

### Participants

**Fig. 1:**
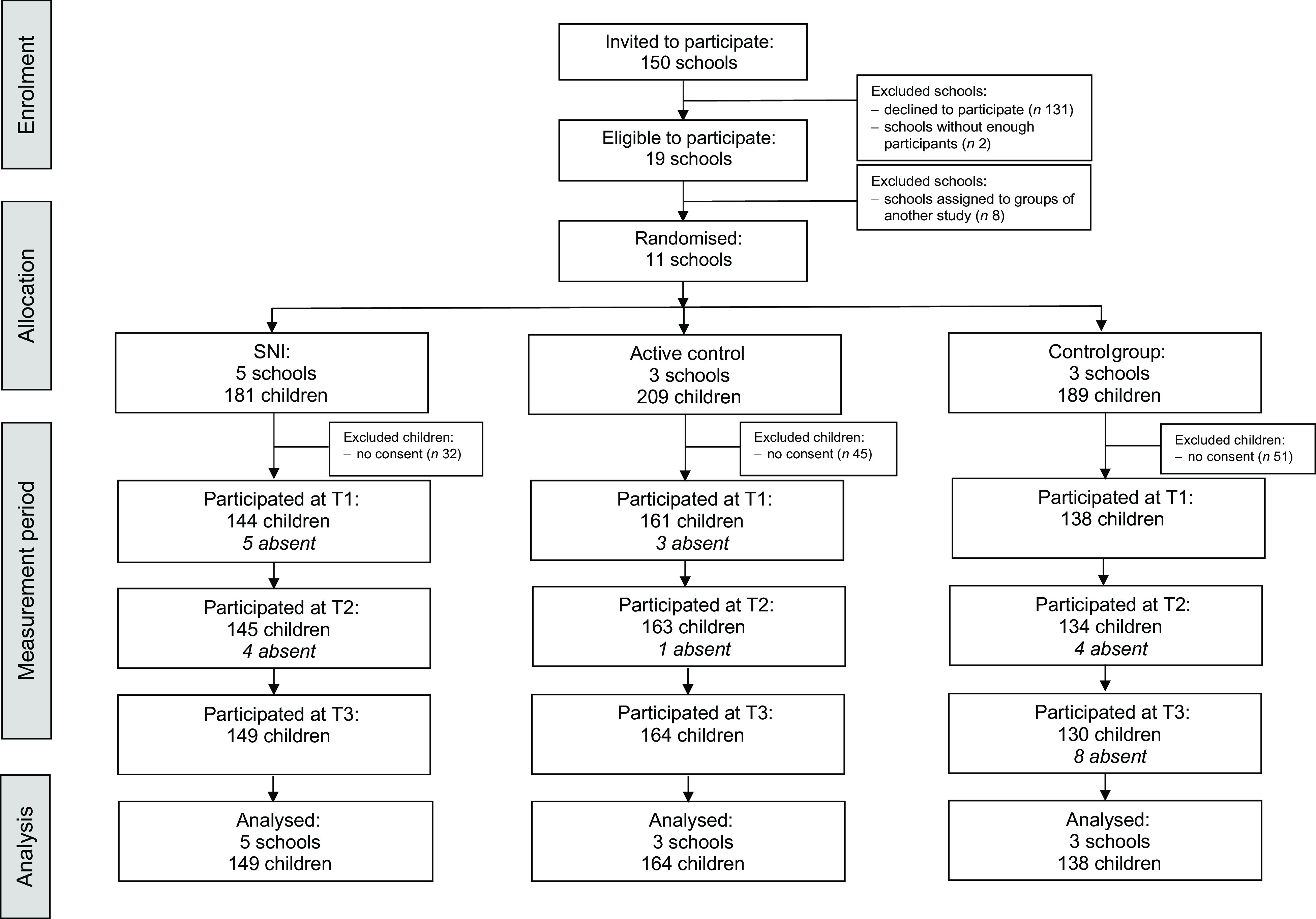
CONSORT flow diagram of study participants. SNI, social network intervention; T1, Time 1; T2, Time 2; T3, Time 3

The study was part of the second data collection phase of the *MyMovez* project^(32)^. Participants were recruited through their school. All schools following a regular education programme were eligible for participation. As shown in Fig. 1, 150 urban and suburban schools in the Netherlands were invited to participate in the second phase of the *MyMovez* project. Twenty-one schools expressed interest in participating; however, two of these schools were unable to participate due to not receiving enough active consents from caregivers as well as children themselves (< 60 % in each classroom^(34)^). Of these nineteen remaining schools, eight schools were assigned to three other conditions from the *MyMovez* project that focused on promoting physical activity^(35)^.

The current study consisted of the eleven schools that were randomly assigned to one of the three conditions that focused on children’s drinking behaviours. Five schools were assigned to the SNI, three to the active control condition and three to the control condition. These schools were located in different areas in the Netherlands, with considerable geographical distance between the conditions (SNI *v*. active control schools ranged from 36 to 203 km; SNI *v*. control schools ranged from 30 to 203 km; active control *v*. control schools ranged from 20 km to 197 km). Therefore, the risk of between-group contamination was negligible. Out of the 579 children in these 11 schools, a total of 128 (22 %) caretakers did not provide consent for their child to participate. Thus, the sample consisted of 451 children (50·8 % girls) between 9 and 14 years old (*M* = 10·74 years; sd = 0·97). Of these children, 149 (47·7 % girls) were allocated to the SNI, 164 (56·1 % girls) to the active control condition and 138 (47·8 % girls) to the control condition (see Fig. 1 for the flow diagram of study participants). The number of participating children was 443 at T1, 442 at T2 and 443 at T3.

#### The social network intervention *Share H*_*2*_ *O*

The SNI involved selecting and training a subset of children from each classroom as influence agents to promote water consumption – as an alternative to SSB – among their peers. The content of the SNI training was nearly the same as the pilot version of the *Share H*_*2*_ *O* intervention^(23)^. However, for this study, we aimed to improve the training content by incorporating more principles of the Self-Determination Theory^(36,37)^ to increase the intrinsic motivation of the peers, in addition to that of the influence agents. Another difference was that in the current study, research assistants were trained to deliver the training to the influence agents, instead of the primary investigator. In general, the purpose of the training was twofold. The first aim was to motivate the influence agents by providing them with the benefits of drinking water – as an alternative for SSB – and encourage them with self-generated arguments to drink more water. The second aim was to support the influence agents in motivating their peers by providing them with the skills to promote water consumption and identifying potential barriers.

Compared to the pilot study, we placed more emphasis in the training on how the influence agents could create an intrinsic motivating climate for their peers while promoting water consumption. Recent research has shown that being intrinsically motivated is an important predictor for positively altering children’s water drinking behaviours^(38)^. According to self-determination theory, being autonomy supportive enhances intrinsic motivation^(37,39,40)^. To this end, possible barriers that the influence agents might encounter while promoting drinking water and how they could overcome these whiles being autonomy supportive were discussed, for example, by taking in consideration the perspective of their peers or providing them with meaningful rationales^(39)^. One week after the training, a follow-up session took place to provide visible support, resolve any issues experienced by the influence agents in their role and refresh the information discussed in the training.

### The active control condition

In the active control condition, children simultaneously received knowledge about the benefits of drinking water–as an alternative for SSB–through a half-hour classroom presentation. This presentation was delivered by research assistants, and the benefits were the same as those discussed in the training of the influence agents. At the time of the presentation, children who had not received consent from their caretakers to participate in the study went to another classroom with the teacher.

### Measures

#### Peer nominations

To identify the influence agents in each classroom, the children were asked to nominate at least one peer whom they ‘wanted to be like’, ‘regarded as good leaders’, ‘went to for advice’^(23,41)^ and ‘talked to about what they drink’^(20)^. For the selection of the influence agents, only same-classroom peer nominations were included. To ensure sex balance in relation to the composition of the classrooms, 15 % of boys and 15 % of girls in the SNI with the most nominations on all items together were selected and trained as influence agents^(23,41)^. On average, five children (sd = 1·06) per participating classroom in the SNI schools were trained as influence agents. This resulted in a total of thirty-seven influence agents from eight classrooms.

#### Water consumption

Children indicated on three different days (i.e. every other day during each data collection wave) how much water they drunk the day before^(23,32,38)^. Response options ranged from 0 = *zero glasses per day* to 7 = *seven or more glasses per day*. An illustration was used to instruct the children that ‘one glass’ also meant one can, bottle, or package of approximately 200 ml. A total score for water consumption was constructed by averaging the children’s reported consumption over the 3 days (Cronbach’s α ranged from 0·65 to 0·78).

#### Sugar-sweetened beverage consumption

Children indicated on three different days (i.e. every other day during each data collection wave) how much sweetened fruit juice, lemonade (based on sugar syrup), soda, energy and sports drinks they drunk the day before^(23,32)^. Response options ranged from 0 = *zero glasses per day* to 7 = *seven or more glasses per day*. The same illustration as with water consumption was used to instruct the children about the portion size. A total score for SSB consumption was constructed by averaging the children’s reported consumption on the five different consumption items over the 3 days (Cronbach’s α ranged from 0·66 to 0·80).

#### Descriptive norms

Children’s perception of the prevalence of their classmates’ behaviour was assessed with the following item: ‘How often do your classmates drink water?’^(32,38)^. Response options ranged from 1 = *never* to 6 = *always*.

#### Injunctive norms

Children’s perception of what their classmates considered appropriate behaviour was assessed with the following item: ‘Do you experience that your classmates think you should drink water?’^(32,38)^. Response options ranged from 1 = *no, certainly not* to 6 = *yes, certainly*.

### Strategy of analyses

Descriptive statistics were calculated to examine the means and standard deviations of all study variables. Subsequently, randomisation checks were performed using one-way ANOVA to test whether there were initial mean-level differences between the conditions for the outcome variables (i.e. water and SSB consumption). Pearson’s correlations were performed for the variables of interest to determine which variable had to be controlled for in the main analyses.

**Fig. 2:**
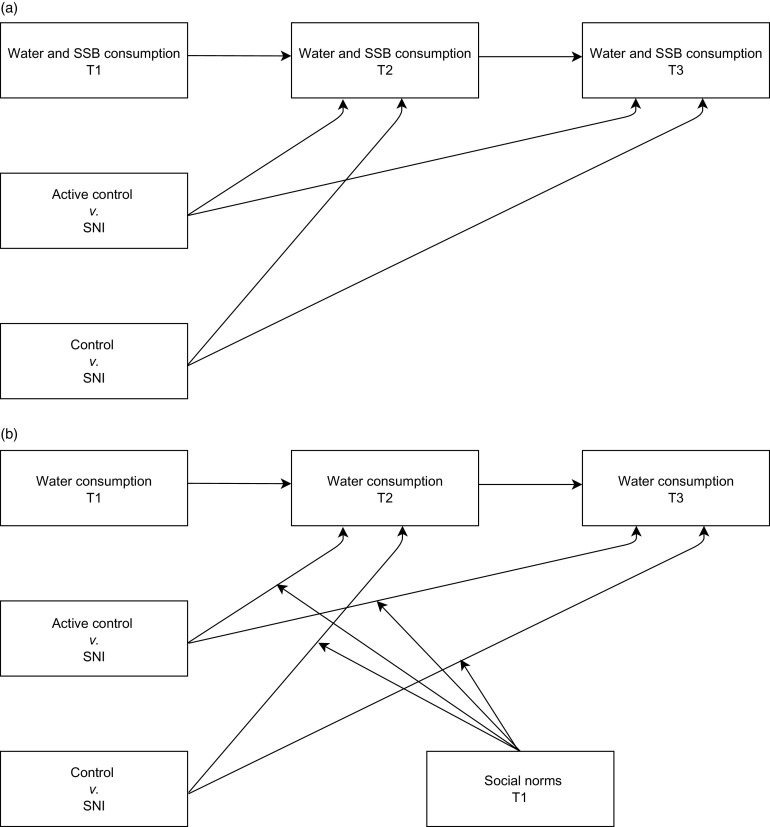
The conceptual models for testing (a) the mean-level differences between conditions on water and SSB consumption after the intervention (T2 and T3), adjusting for previous consumption (T1), and (b) whether prevailing social norms moderated the mean-level differences between conditions on subsequent water consumption (T2 and T3), adjusted for previous consumption (T1); moderation was tested separately for descriptive and injunctive norms; sex was included as a covariate in the first model. SNI, social network intervention; SSB, sugar-sweetened beverage; T1, Time 1; T2, Time 2; T3, Time 3

For the main analyses, three structural path models were tested using Mplus 7.2^(42)^. The first model tested mean-level differences between conditions on water and SSB consumption after the intervention (T2 and T3), adjusting for consumption prior to the intervention (T1; see Fig. 2a); the second model examined whether descriptive norms moderated the mean-level differences between conditions on subsequent water consumption and the third model examined whether injunctive norms moderated the mean-level differences between conditions on subsequent water consumption (see Fig. 2b). In all models, condition was specified as two binary dummy variables with SNI as reference category (coded as 0). In the last two models, the social norm variables were centred prior to creating the interaction terms involving social norms and differences between conditions.

The parameters in the models were estimated applying (full-information) maximum-likelihood estimation with robust standard errors (MLR in Mplus) to account for missing values and potential deviations from multivariate normality. Additionally, the models were adjusted for clustering of the sample – children were ‘nested’ in classrooms – using the Mplus procedure TYPE = COMPLEX, with classroom as the cluster variable. This procedure results in standard errors that are adjusted to account for non-independence within classrooms. The fit of the path models was assessed with the following good fit indices: root mean square error of approximation (RMSEA, with a cut-off value of < 0·08 and *P*-close > 0·05), comparative fit index (CFI, with a cut-off value of > 0·90) and normed *χ*^2^ (*χ*^2^/df, with a cut-off value of < 3·0^(43,44)^). In the structural path analyses, the unstandardised regression coefficient (*b*) provides the estimated mean-level difference between conditions on consumption behaviours following the intervention, adjusted for baseline consumption behaviours. For models yielding significant interaction effects, simple slope analyses^(45)^ were used to examine the regression coefficient of the condition–consumption behaviour relationship across two levels of the moderator (low social norms: –1 sd; high social norms: +1 sd).

## Results

### Preliminary analyses

#### Descriptive statistics and randomisation check

Descriptive statistics showed that on average children consumed 2·99 (sd = 1·70) glasses of water and 0·57 (sd = 0·58) glasses of SSB a day at baseline (T1). The means and standard deviations for all study variables across the conditions are summarised in Table 1. To check whether there were initial mean-level differences between the three conditions on the outcome variables (i.e. water and SSB consumption), one-way ANOVA were conducted. The analyses yielded statistically significant differences at baseline (T1) between conditions for SSB, *F* (2435) = 3·57, *P* = 0·029, but not for water consumption, *F* (2435) = 1·38, *P* = 0·252 (see Table 1). This indicated that the randomisation was not successful for SSB consumption; it is therefore essential to account for these initial differences between conditions to avoid interpreting regression to the mean effects (i.e. groups that have low mean scores are more likely to increase)^(46)^. To account for these initial differences, we included baseline consumption behaviour (T1) as a predictor of consumption behaviour at T2 and T3 in the structural path models.

**Table 1:** Means and standard deviations for all study variables across the conditions per assessment

	SNI (*n* = 149)		Active control (*n* 164)		Control (*n* 138)	
	*M*	sd	*M*	sd	*M*	sd
Water consumption T1	3·03	1·89	3·12	1·58	2·79	1·62
Water consumption T2	3·12	1·99	3·12	1·66	2·76	1·97
Water consumption T3	3·07	2·07	2·29	1·87	2·62	2·00
SSB consumption T1^*^	0·67	0·72	0·49	0·47	0·55	0·58
SSB consumption T2	0·64	0·79	0·45	0·42	0·73	1·06
SSB consumption T3	0·61	0·69	0·57	0·59	0·81	1·21
Descriptive norms T1	3·62	1·03	3·55	1·04	3·74	1·11
Injunctive norms T1	3·69	1·65	3·73	1·65	2·97	1·77

SNI, social network intervention; T1, Time 1; T2, Time 2; T3, Time 3; SSB, sugar-sweetened beverage.

* The three conditions differed significantly on this variable (*P* < 0·05).

#### Correlations among variables

Pearson’s correlations were computed to examine the bivariate relationship between the variables of interest (see Table 2). Children’s water consumption was positively related to descriptive norms and not related to injunctive norms. Children’s SSB consumption was only negatively related to sex, indicating that boys drank more SSB than girls. Therefore, we included sex as a covariate in the model testing the mean-level differences between conditions on SSB consumption.

**Table 2:** Correlations among all study variables (*n* 451)

	1	2	3	4	5	6	7	8
1-Sex*								
2-Water consumption T1	0·01							
3-Water consumption T2	0·03	0·48§						
4-Water consumption T3	0·01	0·39§	0·56§					
5-SSB consumption T1	–0·16‡	0·01	–0·05	–0·06				
6-SSB consumption T2	–0·17‡	–0·04	0·03	0·01	0·46§			
7-SSB consumption T3	–0·09	–0·06	0·03	–·03	0·32§	0·55§		
8-Descriptive norms T1	0·02	0·12†	0·10†	0·08	–0·00	–0·09	0·03	
9-Injunctive norms T1	0·02	0·02	0·01	–0·02	0·03	–0·02	0·00	0·09

T1, Time 1; T2, Time 2; T3, Time 3.

* 0 = boy and 1 = girl.

† *P* < 0·05.

‡ *P* < 0·01.

§ *P* < 0·001.

### Main analyses

#### Condition differences on changes in water and sugar-sweetened beverage consumption

The first structural path model examined whether children exposed to the SNI increased their water and decreased SSB consumption compared to those in the active control condition (H1a and H2a) and control condition (H1b and H2b). This model demonstrated a good fit to the observed data, RMSEA = 0·04, CFI = 0·97 and normed *χ*^2^ = 1·58. Table 3 presents the results of this model. The model showed that there was a significant mean-level difference between the SNI and control condition on SSB consumption at T2, adjusting for baseline SSB consumption (*b* = 0·20, se = 0·10, *β =* 0·25, *P* = 0·035, 95 % CI [0·02, 0·48]). This indicated that, immediately after the intervention, children exposed to the SNI consumed an average of 0·20 glasses less SSB per day than those in the control condition, adjusting for SSB consumption prior to the intervention. At T3, there was a marginally significant mean-level difference between the SNI and active control condition on SSB consumption, adjusted for baseline SSB consumption (*b* = 0·17, se = 0·10, *β =* 0·20, *P* = 0·061, 95 % CI [–0·01, 0·40]). This indicated that there was a trend showing that, 4 weeks after the start of the intervention, children exposed to the SNI consumed an average of 0·17 glasses less SSB per day than children in the active control condition (adjusting for SSB consumption at T1). For water consumption, there were no statistically significant differences between the three conditions.

**Table 3:** Results for the model testing mean-level differences between conditions on water and SSB consumption after the intervention (*n* 451)

	Water consumption					SSB consumption				
	*b*	se	*β*	*P*	CI	*b*	se	*β*	*P*	CI
Regression paths										
Active control [1] *v*. SNI [0] – Behaviour T2	–0·02	0·18	–0·01	0·936	−0·20, 0·19	–0·03	0·06	–0·04	0·633	−0·18, 0·11
Control [1] *v*. SNI [0] – Behaviour T2	–0·17	0·31	–0·09	0·589	−0·43, 0·24	0·20	0·10	0·25	**0·035**	0·02, 0·48
Active control [1] *v*. SNI [0] – Behaviour T3	–0·31	0·25	–0·16	0·217	−0·40, 0·09	0·17	0·10	0·20	0·061	−0·01, 0·40
Control [1] *v*. SNI [0] – Behaviour T3	–0·20	0·20	–0·10	0·308	−0·29, 0·09	0·19	0·13	0·21	0·131	−0·06, 0·49
Stability paths										
Behaviour T1 – Behaviour T2	0·51	0·06	0·47	< **0·001**	0·36, 0·58	0·51	0·06	0·49	< **0·001**	0·32, 0·66
Behaviour T2 – Behaviour T3	0·62	0·05	0·58	< **0·001**	0·51, 0·64	0·62	0·05	0·64	< **0·001**	0·46, 0·82
Control variables										
Sex* – Behaviour T2	–0·00	0·16	–0·00	0·977	−0·08, 0·08	–0·00	0·16	–0·08	0·119	−0·18, 0·02
Sex* – Behaviour T3	0·02	0·24	0·01	0·923	−0·11, 0·12	0·02	0·24	–0·03	0·431	−0·10, 0·04

SSB, sugar-sweetened beverage; *b*, unstandardised regression coefficient estimating the mean-level difference between conditions, adjusted for previous consumption; *β*, standardised regression coefficient; SNI, social network intervention; T1, Time 1; T2, Time 2; T3, Time 3.

* 0 = boy, 1 = girl; boldface indicates statistical significance (*P* < 0·05); numbers in parentheses represent the binary dummy-coded values; SNI is the reference category in the model.

### Moderating effects of norms on water consumption

#### Descriptive norms

**Fig. 3:**
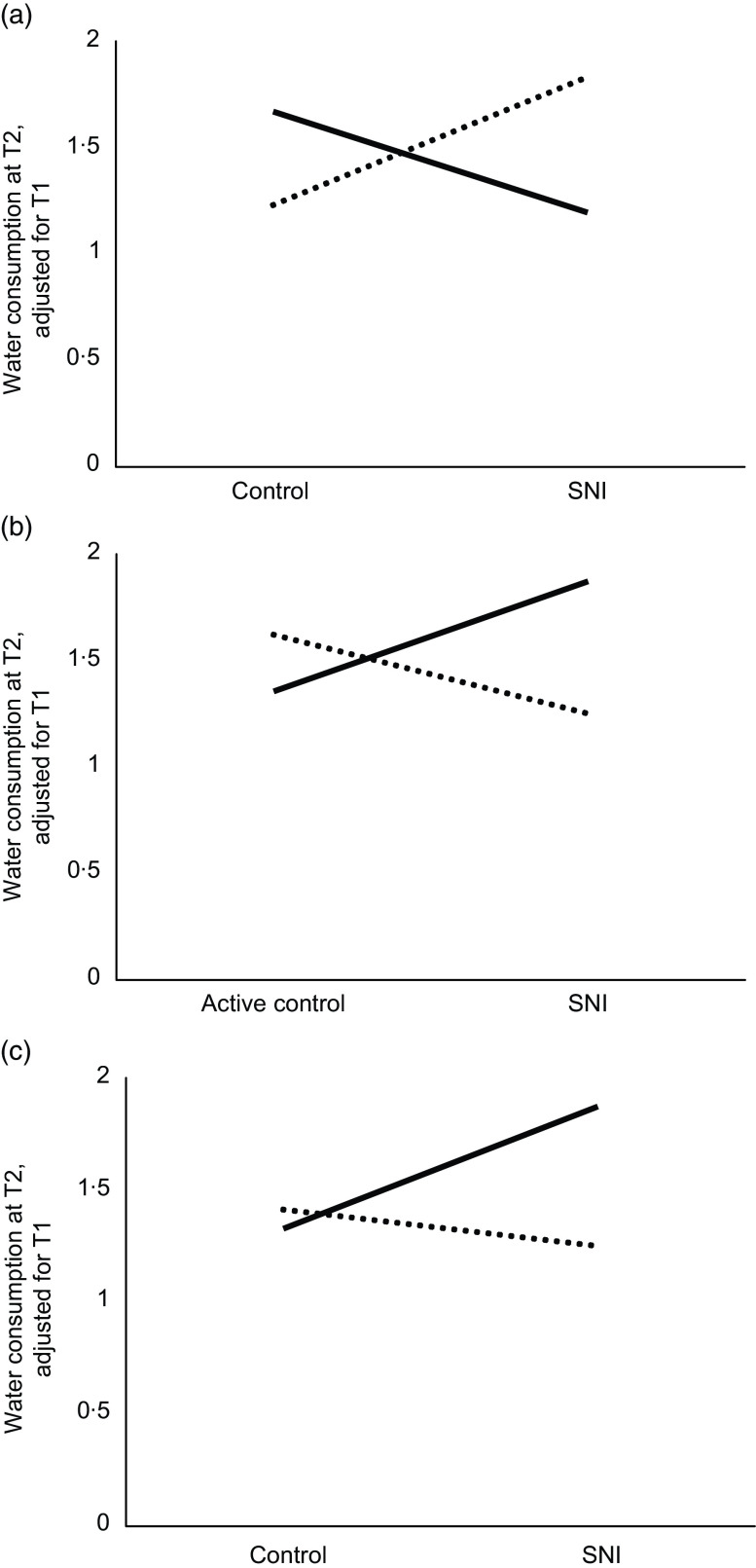
The interactions between descriptive norms (a) or injunctive norms (c and b) and conditions on water consumption at T2, adjusted for T1 consumption. T1, Time 1; T2, Time 2. 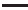, Low; 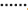, High

The second structural path model examined the potential moderating role of descriptive norms on the effectiveness of the SNI. This model showed a good fit to the observed data, RMSEA = 0·02, CFI = 0·99 and normed *χ*^2^ = 1·16. At T2, the main effect of descriptive norms emerged as statistically significant, but this effect was qualified by a significant interaction between descriptive norms and the difference among the SNI and control conditions on water consumption, adjusting for water consumption at T1 (*b* = –0·38, se = 0·16, *β =* –0·12, *P* = 0·028, 95 % CI [–0·23, –0·01]; see Table 4). To interpret this interaction, we conducted simple slope analysis. Fig. 3a presents the significant interaction, with water consumption at T2 (adjusted for T1) on the *y*-axis, conditions on the *x*-axis and separate regression lines for participants with high (+1 sd) and low (–1 sd) descriptive norms. This figure indicates that there was a positive relation between conditions and water consumption at T2 (adjusted for T1) for high descriptive norms (*b* = –0·60, se = 0·42, *P* = 0·154) and a negative relation for low descriptive norms (*b* = 0·25, se = 0·29, *P* = 0·381), but neither slope significantly differed from zero. Thus, there is some evidence to suggest that children reporting higher descriptive norms consumed more water in the SNI and less water in the control condition compared to those with lower norms. While the simple slopes differed in valence, this interpretation is made cautiously considering the lack of statistically significant simple slopes.

**Table 4:** Results for the model testing descriptive norms as a moderator of the mean-level differences between conditions on water consumption (*n* 451)

	Water consumption				
	*b*	se	*β*	*P*	CI
Regression paths					
Active control [1] *v*. SNI [0] – Behaviour T2	–0·00	0·19	–0·00	0·982	−0·20, 0·20
Control [1] *v*. SNI [0] – Behaviour T2	–0·18	0·32	–0·10	0·584	−0·43, 0·24
Descriptive norms T1 – Behaviour T2	0·23	0·11	0·13	**0·048**	0·00, 0·25
Active control [1] *v*. SNI [0] X Descriptive norms T1 – Behaviour T2	–0·13	0·13	–0·04	0·308	−0·13, 0·04
Control [1] *v*. SNI [0] X Descriptive norms T1 – Behaviour T2	–0·38	0·16	–0·12	**0·028**	−0·23, -0·01
Active control [1] *v*. SNI [0] – Behaviour T3	–0·31	0·24	–0·16	0·195	−0·40, 0·08
Control [1] *v*. SNI [0] – Behaviour T3	–0·22	0·19	–0·11	0·249	−0·30, 0·08
Descriptive norms T1 – Behaviour T3	–0·04	0·21	–0·02	0·844	−0·24, 0·19
Active control [1] *v*. SNI [0] X Descriptive norms T1 – Behaviour T3	0·06	0·27	0·02	0·830	−0·14, 0·18
Control [1] *v*. SNI [0] X Descriptive norms T1 – Behaviour T3	0·23	0·28	0·07	0·399	−0·09, 0·23
Stability paths					
Behaviour T1 – Behaviour T2	0·49	0·06	0·45	< **0·001**	0·34, 0·56
Behaviour T2 – Behaviour T3	0·62	0·05	0·58	< **0·001**	0·50, 0·65

*b*, unstandardised regression coefficient estimating the mean-level difference between conditions, adjusted for previous consumption; *β*, standardised regression coefficient; SNI, social network intervention; T1, Time 1; T2, Time 2; T3, Time 3; boldface indicates statistical significance (*P* < 0·05); numbers in parentheses represent the binary dummy-coded values; SNI is the reference category in the model.

#### Injunctive norms

The last structural path model examined the potential moderating role of injunctive norms on the effectiveness of the SNI. This model showed a good fit to the observed data, RMSEA = 0·06, CFI = 0·99 and normed *χ*^2^ = 2·65. At T2, the main effect of injunctive norms emerged as statistically significant, but this effect was qualified by a significant interaction effect between injunctive norms and the difference among the SNI and active control conditions on water consumption, adjusting for water consumption at T1 (*b* = 0·26, se = 0·12, *β* = 0·14, *P* = 0·050, 95 % CI [–0·00, 0·28]). There was also a significant interaction effect between injunctive norms and the difference among the SNI and control conditions on water consumption, adjusting for water consumption at T1 (*b* = 0·21, se = 0·11, *β* = 0·11, *P* = 0·050, 95 % CI [0·00, –0·22]; see Table 5). To examine these significant interactions, we conducted simple slope analysis. Figs 3b and c present the significant interaction, with water consumption at T2 (adjusted for T1) on the *y*-axis, conditions on the *x*-axis and separate regression lines for participants with high (+1 sd) and low (–1 sd) injunctive norms. Fig. 3b indicates that there was a positive relation between conditions and water consumption at T2 (adjusted for T1) for low injunctive norms (*b* = –0·52, se = 0·26, *P* = 0·044) and a negative relation for high injunctive norms (*b* = 0·37, se = 0·29, *P* = 0·206). Fig. 3c also indicates that there was a positive relation between conditions and water consumption at T2 (adjusted for T1) for low injunctive norms (*b* = –0·54, se = 0·28, *P* = 0·056) and a negative relation for high injunctive norms (and *b* = 0·16, se = 0·44, *P* = 0·710), but neither slope significantly differed from zero. Thus, these interactions collectively suggest that children reporting lower injunctive norms consumed more water in the SNI condition and less water in the active control condition and control condition compared to those with higher norms.

**Table 5:** Results for the model testing injunctive norms as a moderator of the mean-level differences between conditions on water consumption *(n 451)*

	Water consumption				
	*b*	se	*β*	*P*	CI
Regression paths					
Active control [1] *v*. SNI [0] – Behaviour T2	–0·07	0·18	–0·02	0·685	–0·11, 0·07
Control [1] *v*. SNI [0] – Behaviour T2	–0·19	0·33	–0·05	0·564	–0·21, 0·11
Injunctive norms T1 – Behaviour T2	–0·18	0·09	–0·17	**0·039**	–0·33, -0·01
Active control [1] *v*. SNI [0] X Injunctive norms T1 – Behaviour T2	0·26	0·12	0·14	**0·050**	–0·00, 0·28
Control [1] *v*. SNI [0] X Injunctive norms T1 – Behaviour T2	0·21	0·11	0·11	**0·050**	0·00, 0·22
Active control [1] *v*. SNI [0] – Behaviour T3	–0·27	0·24	–0·07	0·267	–0·18, 0·05
Control [1] *v*. SNI [0] – Behaviour T3	–0·23	0·21	–0·05	0·277	–0·15, 0·04
Injunctive norms T1 – Behaviour T3	0·06	0·04	0·05	0·122	–0·01, 0·03
Active control *v*. SNI [0] X Injunctive norms T1 – Behaviour T3	–0·13	0·09	–0·06	0·163	–0·15, 0·03
Control [1] *v*. SNI [0] X Injunctive norms T1 – Behaviour T3	–0·13	0·08	–0·07	0·095	–0·14, 0·01
Stability paths					
Behaviour T1 – Behaviour T2	0·51	0·06	0·47	< **0·001**	0·36, 0·58
Behaviour T2 – Behaviour T3	0·62	0·05	0·57	< **0·001**	0·50, 0·65

*b*, unstandardised regression coefficient estimating the mean-level difference between conditions, adjusted for previous consumption; *β*, standardised regression coefficient; SNI, social network intervention; T1, Time 1; T2, Time 2; T3, Time 3; boldface indicates statistical significance (*P* < 0·05); numbers in parentheses represent the binary dummy-coded values; SNI is the reference category in the model.

## Discussion

The SNI *Share H*_*2*_ *O* aimed to positively alter children’s healthy drinking behaviours by exposing them to influence agents from their own classroom who promoted water consumption as an alternative to SSB. The current study tested the effectiveness of this approach by comparing it to an active control condition – based on the principles of mass media campaigns – and a control condition without any intervention. Furthermore, the moderating role of the prevailing social norms in the context was tested. The findings showed that children exposed to the SNI *Share H*_*2*_ *O* consumed less SSB afterwards compared to children in the active control condition and control condition. No differences between the conditions were found for water consumption. However, the effectiveness of the SNI on water consumption seems to depend on the prevailing social norms. More specifically, children exposed to the SNI with initially higher perceived descriptive norms and lower perceived injunctive norms consumed more water afterwards compared to those in the active control condition and the control condition.

Our findings regarding the effect on SSB consumption showed that after the intervention, children exposed to the SNI remained stable in their SSB consumption, while the children in the active control condition and control condition consumed more SSB. This finding is different compared to our previous pilot studies in which children exposed to the SNI decreased in their SSB consumption over time^(22,23)^. A possible explanation may lie in seasonal differences. In the current study, the baseline measurement took place during the winter, while the intervention took place during the spring, which resulted in much weather difference between the two measurements. In the previous pilot studies^(22,23)^, both measurements took place in the same season and the weather was therefore more stable. Thus, it may be that the SNI with influence agents spreading the message or behaviour in their peer group *prevented* children from turning to SSB during warm weather. However, future research is needed to explore this possibility, along with replication studies over the years to shed more light on this reasoning.

Nevertheless, it is in line with our expectations that when peers communicate about the benefits of drinking water – as an alternative for SSB – it could be an effective strategy to prevent children from consuming SSB. This effect was found on the short term and compared to the control condition without an intervention. However, the question remains why the difference between the SNI and the active control is not so pronounced. It could be that the benefits of drinking water presented to the children in the active control condition were convincing enough for the children, even when the research assistants communicated them. These benefits were formulated based on short-term outcomes (e.g. ‘Drinking water helps you concentrate better at school’) as they are generally considered to be more motivating than long-term consequences^(57)^. It is therefore possible that the framing of these messages itself was already strong and convincing, irrespective of the sender. However, the findings suggest that when these benefits are communicated by peers, the effects are less short-lived, given that a marginal difference was found between the SNI and the active control condition at T3. Nevertheless, more research is needed to further investigate this. Altogether, the findings of this study suggest that the SNI *Share H*_*2*_ *O* can be fruitful for schools specifically targeting SSB consumption.

Contrary to our expectations and a previous pilot study^(23)^, we did not find that the SNI was effective in increasing water consumption in general. One reason for this finding could be that the general opinion about water drinking has changed in the past years. Our pilot study was conducted 4 years ago, and meanwhile, a great deal of (media) attention has been paid to the health benefits of drinking water, including the environmental consequences of drinking SSB instead of water (i.e. plastic soup). For example, by the national organisation *Jongeren Op Gezond Gewicht* [*Youth at a Healthy Weigh*t]^(51,52)^ that focuses on changing the water drinking norms in schools. The plastic soup also received a lot of (inter)national attention, for example, from the World Wildlife Fund, and even a famous national children’s choir released a song called ‘Plastic Soep*’* [Plastic Soup] in 2017, which became very popular^(53,54)^. This (media) attention for water consumption has probably inspired some children and parents to drink more water in recent years. For this group, the content of the *Share H*_*2*_ *O* intervention – which mainly focuses on the benefits of drinking water – could have been less or perhaps even not inspiring at all. It is therefore essential that future research focuses on updating the content in order to better respond to the current consumption behaviour, norm and knowledge of the target children. This can be achieved, for example, by involving these children in the development of the content (i.e. co-design^(55)^) and thus taking into account their vision, which can increase intrinsic motivation in health interventions^(56)^. Recent research has shown that intrinsic motivation is a crucial predictor of changing children’s water consumption^(38)^.

In line with our expectations, we indeed found that the prevailing social norms concerning water drinking moderated the effectiveness of the SNI on water consumption. First, the SNI intervention was found to be more effective among children who already perceived that their classmates were drinking water before the intervention started (i.e. higher perceived descriptive norm). Probably, the higher prevalence of water drinking peers in their environment led these children to consider water drinking as a normal and socially acceptable behaviour. When water drinking was promoted by peers they wanted to be like or went to for advice, this intervention ‘message’ was congruent with what these children were already perceiving in their environment. This may have resulted in it being perceived as a familiar message, making it easier to adjust their behaviour accordingly. In contrast, for children with initially lower perceived descriptive norms, it may be that the discrepancy between the ‘message’ (i.e. drink more water) and what they perceived in their environment may have been too large to bridge, leading to lower behavioural change. This reasoning is consistent with the contextual-congruence model which suggests that higher levels of congruence between values, beliefs and behaviours across children’s social environments facilitate the internalisation process^(47)^. This may also play a role between the social environment and intervention messages, as the lack of incongruent talk about the target behaviour in the social environment is a facilitative condition of media effects^(48)^.

Second, we found that children who initially perceived lower injunctive peer norm consumed more water after being exposed to the SNI, while children who perceived higher levels of injunctive norm in their environment did not change their water consumption. More specifically, the intervention was not successful among children who perceived that their classmates thought that they should drink water. Previous research has shown that higher levels of injunctive norm can be perceived as a coercive pressure from others to conduct the target behaviour^(27)^. Thus, it could be that in a context without this perceived peer pressure to drink water (i.e. low levels of injunctive norm), children may become more motivated^(40)^ to adopt their behaviour in accordance with the water promoting message in the SNI. In contrast, in a context where they beforehand do perceive high levels of peer pressure to drink water (i.e. high levels of injunctive norm), they could become less motivated to adopt their behaviour in accordance with the message.

It is important to underline that the current study yielded a conflicting pattern compared to the previous pilot study examining the moderating role of injunctive norms in SNI^(22)^. More specifically, the study of Franken et al. found that children who initially perceived higher injunctive peers norms were more likely to change their behaviour^(22)^. A possible reason for this conflicting pattern could be that the Franken et al. study was conducted on a Caribbean island involving cultural differences regarding social norms and energy intake-related behaviours^(49)^. Further research is therefore needed to determine how exactly the prevailing social norms in the context interacts with the effectiveness of SNI. Additionally, the next step for future research is also to examine whether and how SNI change the perceived social norms of children, which in turn may cause the intervention effect. Previous research showed that changes in students’ perceptions of descriptive drinking norms mediated the effect of brief motivational interventions targeting alcohol consumption^(50)^.

### Limitations and future research

This study had a number of strengths, including a relatively large sample, multiple time points and a theoretically well-founded intervention. However, some limitations need to be addressed in interpreting the findings. First, the assessment of children’s drinking behaviours was based on self-report. Although self-reported intake is usually considered reliable^(58)^, one should keep in mind that there is potential for under-reporting or over-reporting of these behaviours. Future studies could try to replicate our findings using additional and more direct measurements of beverage consumption, such as observations at school or flow meters attached to the schools’ water fountains^(59)^. Second, we only measured the effect immediately after the intervention and 4 weeks later. Although our results indicated some improvements in children’s drinking behaviours at least 4 weeks after the intervention, the next step is to replicate this study and include a follow-up assessment 1 year later to examine the effect on the longer term^(15)^.

Third, the current study solely focused upon stimulating peer influence and did not consider other important social influences. Despite the fact that peers are increasingly important during childhood^(11)^, parents continue to exert influence^(60)^. Recent research has shown that parental norms also play an important role in changing children’s healthy drinking behaviours^(38)^. Hence, a conceivable approach to improve the SNI could be to not merely incorporate peer influence but additionally motivate parents to set a good example at home for their children with regard to water drinking^(61)^.

## Conclusion and implications

The findings of this study support the growing body of SNI research demonstrating that utilising the strong influences of peers seems to strengthen interventions promoting healthy behaviours^(13,22,23)^. Selecting influencing agents and motivating them to drink (more) water and to spread this message and behaviour among their peers could prevent children from consuming more SSB. In addition, the study emphasises that the success of the SNI *Share H*_*2*_ *O* on water consumption depends on the prevailing peer norms in the context in which it is implemented^(26,29–31)^. The current research focused on children’s drinking behaviour, but this social network approach, which makes use of the strong influence of peers^(11)^ and focuses on increasing the motivation of children^(38)^, might also have fruitful effects for other consumption behaviours, such as increasing the intake of healthy snacks.
